# RAD51AP1 promotes progression of ovarian cancer via TGF‐β/Smad signalling pathway

**DOI:** 10.1111/jcmm.15877

**Published:** 2020-12-13

**Authors:** Hongyu Zhao, Yan Gao, Qi Chen, Jie Li, Meng Ren, Xiaoting Zhao, Wentao Yue

**Affiliations:** ^1^ Central Laboratory Beijing Obstetrics and Gynecology Hospital Capital Medical University Capital Medical University Beijing China

**Keywords:** mutation, ovarian cancer, prognostic, progression, RAD51AP1

## Abstract

Ovarian cancer (OC) is one of the leading causes of female deaths. However, the molecular pathogenesis of OC has still remained elusive. This study aimed to explore the potential genes associated with the progression of OC. In the current study, 3 data sets of OC were downloaded from the GEO database to identify hub gene. Somatic mutation data obtained from TCGA were used to analyse the mutation. Immune cells were used to estimate effect of the hub gene to the tumour microenvironment. RNA‐seq and clinical data of OC patients retrieved from TCGA were used to investigate the diagnostic and prognostic values of hub gene. A series of in vitro assays were performed to indicate the function of hub gene and its possible mechanisms in OC. As a result, RAD51AP1 was found as a hub gene, which expression higher was mainly associated with poor survival in OC patients. Up‐regulation of RAD51AP1 was closely associated with mutations. RAD51AP1 up‐regulation accompanied by accumulated Th2 cells, but reduced CD4 + T cells and CD8 + T cells. Nomogram demonstrated RAD51AP1 increased the accuracy of the model. Down‐regulation of RAD51AP1 suppressed proliferation, migration and invasion capabilities of OC cells in vitro. Additionally, scatter plots showed that RAD51AP1 was positively correlated with genes in TGF‐β/Smad pathway. The above‐mentioned results were validated by RT‐qPCR and Western blotting. In conclusion, up‐regulation of RAD51AP1 was closely associated with mutations in OC. RAD51AP1 might represent an indicator for predicting OS of OC patients. Besides, RAD51AP1 might accelerate progression of OC by TGF‐β/Smad signalling pathway.

## 1 INTRODUCTION

Ovarian cancer (OC), one of the most fatal and aggressive tumours of the female reproductive system, has emerged with an increased incidence in recent years.^1^ Due to confusing symptoms and no screening for early detection, the 5‐year overall survival (OS) was reported as high as about 45%. Early diagnosis was correlated with improved OS, while poor OS was associated with late diagnosis and high rate of recurrence.^2^, ^3^ However, the evolutionary mechanisms of OC have still remained elusive. Thus, it is crucial to understand the precise molecular mechanisms involved in the carcinogenesis, proliferation, invasion of OC and develop further effective diagnostic and therapeutic methods for managing OC.^4^, ^5^, ^6^

In recent years, the microarray and high‐throughput sequencing technologies have rapidly advanced to screen the genetic alterations in genome level and explore further potential biomarkers.^7^, ^8^ A number of databases are available from free public gene expression data repositories, such as The Cancer Genome Atlas (TCGA) project and the Gene Expression Omnibus (GEO) database. Numerous studies employed these databases and different bioinformatics methods to seek novel markers and molecular mechanisms for OC.^9^, ^10^

RAD51‐dependent homologous recombination (RAD51AP1) is a RAD51 accessory protein that specifically stimulates joint molecule formation through the combination of structure‐specific DNA binding and physical contact with RAD51, and knockdown of RAD51AP1 results in increase of sensitivity to DNA damaging agents and impaired HR.^11^ In the present study, 3 data sets of OC were downloaded from the GEO database to identify RAD51AP1 as a hub gene in OC. A variety of bioinformatics methods and experimental assays were utilized to understand the biological functions and relative molecular mechanism underlying carcinogenesis. To our acknowledge, this study initially explored the relationship of RAD51AP1 and gene mutations and tumour microenvironment (TME), then established a RAD51AP1‐related nomogram to investigate patients’ survival, and demonstrated that RAD51AP1 might accelerate progression of OC by TGF‐β/Smad signalling pathway.

## 2 METHODS

### 2.1 OC and normal controls data sets

Data retrieved from multiple researches were used for integrated analysis in this study, including data from TCGA project and GEO database. RNA‐seq data and corresponding clinicopathological data of OC patients in TCGA were obtained from UCSC Xena (https://xenabrowser.net/datapages/). The clinicopathological characteristics included age, pharmaceutical therapy, stage, grade, outcome, follow‐up, etc The somatic mutation status for OC (workflow type: VarScan2 Variant Aggregation and Masking) was obtained from TCGA (https://portal.gdc.cancer.gov/repository). The microarray data sets (GSE14001, GSE40595 and GSE54388) were downloaded from GEO database (https://www.ncbi.nlm.nih.gov/) and were annotated according to the platform of Affymetrix HG‐U133 Plus 2.0 (GPL570).

Raw microarray data were downloaded and normalized using a robust multi‐array average (RAM) method using ‘affy’ package in R software for estimation of missing values, background correction, log2 transformation, quantile normalization and data summarization. The GSE14001 data set included 3 normal samples and 20 OC samples; GSE40595 contained 6 normal samples and 32 OC samples; GSE54388 composed of 6 normal samples and 16 OC samples.

### 2.2 Cell culture and siRNA transfection

In the present research, OC cell lines (HEY, SKOV3) were obtained from American Type Culture Collection (ATCC). All cell lines were cultured in Roswell Park Memorial Institute (RPMI)‐1640 medium (Gibco, Gaithersburg, MD, USA) supplemented with 10% foetal bovine serum (FBS; HyClone Laboratories Inc, Logan, UT, USA) and 100 U/ml penicillin/streptomycin (Gibco, Gaithersburg, MD, USA) at 37°C in an atmosphere containing 5% CO2. All cell lines were transfected with Lipofectamine™ RNAmax (Invitrogen, Carlsbad, CA, USA). RAD51AP1 target‐specific small interfering RNA (siRNA) was synthesized by JTSBIO Co., Ltd. (Wuhan, China). The sequences of RAD51AP1 target‐specific siRNA (si‐RAD51AP1) were as follows: siRNA1, 5’‐GCCUGUGAGACAUAAGAAATTUUUCUUAUGUCUCACAGGCTT‐3’; siRNA2, 5'‐ GUCUCCUCAUAUCUCUAAUTTAUUAGAGAUAUGAGGAGACTT‐3'; siRNA3, 5'‐ CAGAUUAGCACGAGUUAAATTUUUAACUCGUGCUAAUCUGTT‐3', and the sequence of control was 5'‐UUCUCCGAACGUGUCAGGUTTUCCAGGTCUAGTT‐3'. HEY and SKOV3 cells were cultured in 6‐well plates. Cells were transfected with 100 nmol/L RAD51AP1 siRNA or control siRNA. The cells transfected siRNA were incubated for 24 hours for subsequent assays, including quantitative reverse transcription polymerase chain reaction (RT‐qPCR), proliferation, wound healing and transwell migration. In addition, the OC cells are transfected with siRAD51AP1 or siControl for 4‐6 hours, and then, the cells are cultured with RPMI‐1640 medium supplemented without FBS for 24 hours; subsequently, cells transfected with siRAD51AP1 are incubated with TGFβ for 48 hours for subsequent assays.

### 2.3 RNA extraction and RT‐qPCR

Total RNA was isolated using TRIzol reagent according to the manufacturer's instructions (Invitrogen, Carlsbad, CA). Synthesis of cDNA was performed by using the ReverTra Ace qPCR RT kit (Toyobo, Shanghai, China). The RT‐qPCR was carried out through an ABI 7500 Real‐Time PCR system (Applied Biosystems, Foster City, USA) using the SYBR Premix EX Taq™ (Takala, Dalian, China). The primer sequences are listed in Table S1. The expression of glyceraldehyde‐3‐phosphate dehydrogenase (GAPDH) served as internal standard. Relative gene expression was determined by using the 2^−ΔΔCT^ comparative method.

### 2.4 Western blot analysis

Total protein was extracted from SKOV3 and HEY after transfection and lysed using radio‐immunoprecipitation assay (RIPA) buffer (Thermo Fisher Scientific, Waltham, MA, USA) supplemented with protease inhibitor cocktail (Roche Diagnostics, Basel, Switzerland). Total protein amount was measured using bicinchoninic acid (BCA) assay (Thermo Fisher Scientific, Waltham, MA, USA). 30 μg of proteins per sample was separated by 10% sodium dodecyl sulphate‐polyacrylamide gel electrophoresis (SDS‐PAGE) (Gene Molecular Biotech Inc, Shanghai, China), transferred onto polyvinylidene difluoride (PVDF) membranes (Gene Molecular Biotech, Inc, Shanghai, China) and blocked with 5% skimmed milk for 1 hours at room temperature. Subsequently, the PVDF membranes were then incubated in 0.5% bovine serum albumin (BSA) overnight at 4°C with primary antibodies (Cell Signaling Technology, Inc, Danvers, MA, USA) at the following dilutions: rabbit anti‐GAPDH (dilution, 1:1000), rabbit anti‐total Smad 2/3 (dilution, 1:1000), rabbit anti‐phospho‐Smad2 (dilution, 1:1000), rabbit anti‐phospho‐Smad3 (dilution, 1:1000), rabbit anti‐Vimentin (dilution, 1:1000). After that, the membranes were incubated with horseradish peroxidase (HRP)‐conjugated IgG secondary antibodies (dilution, 1:7500; Cell Signaling Technology, Inc, Danvers, MA, USA) for 1 hours. Next, the expression levels were detected by an enhanced chemiluminescent (ECL) kit (Roche Diagnostics, Basel, Switzerland) via Western blot imaging system.

### 2.5 Cell proliferation assay

Here, 1 × 10^3^ cells were incubated in 96‐well plates. Then, 10 µL Cell Counting Kit‐8 (CCK‐8; Dojindo, Rockville, MD, USA) solution was added to each well and incubated for 2 hours for evaluating cell proliferation. The absorbance of each well was measured at wavelength of 450 nm using a Tecan Infinite M1000 PRO (Tecan, Switzerland) from days 1 to 4.

### 2.6 Cell wound healing assay

Cell migration was evaluated with wound healing assay. 1 × 10^6^ SKOV3 cells were seeded on 6‐well plates. Wound was produced by a 100 μL pipette tube. Images were then captured after three times washing with phosphate‐buffered saline (PBS), and the exchanging medium (RPMI‐1640 medium) was added into the plate. The spread of wound was observed after 0 and 96 hours. Cell migration was photographed in several pre‐marked spots with a microscope and quantified by ImageJ software.

### 2.7 Transwell migration and invasion assays

The transwell migration and invasion assays were conducted in 24‐well plates (Corning, Inc, NY, USA). For the transwell migration assay, 1.5 × 10^4^ cells suspended in 50μL serum‐free RPMI −1640 were placed in the upper chambers and 600 μL RPMI −1640 containing 10% FBS were filled in the lower compartment. The cells were incubated at 37°C for 24 hours. The successfully translocated cells were fixed with 4% paraformaldehyde (PFA) and stained with 0.1% crystal violet and counted in four randomly chosen fields (×200) under a microscope. For the transwell invasion assay, 1.2 × 10^5^ cells were seeded on transwells coated with 50 μL Matrigel (dilution of 1:4 with 0.2% BSA) (Sigma‐Aldrich, St. Louis, MO, USA). The culture conditions were the same as described for the transwell migration assay. After 48 hours, the cells on the lower surface were fixed, stained and photographed microscopically.

### 2.8 Statistical analysis

#### 2.8.1 Identification and functional annotation of differentially expressed genes (DEGs)

We merged the three data sets to one data set, and then, key DEGs were identified by using a normalization method via ‘sva’ package in R 3.5.0 software. Adjusted *P* < 0.01 and |log FC|> 2 were regarded as cut‐off values. Gene Ontology (GO) and Kyoto Encyclopedia of Genes and Genomes (KEGG) pathway enrichment analyses were performed by clusterProfiler package.^12^ The ontology included three categories: biological process (BP), molecular function (MF) and cellular component (CC). *P* < 0.05 was defined as cut‐off value in this analysis.

#### 2.8.2 Construction of protein‐protein interaction (PPI) network

The STRING database (ver. 10.5, https://string‐db.org/) was used to construct PPI network in DEGs. A combined score >0.4 was taken as inclusion criterion into account. The visualization of PPI network was realized by Cytoscape 3.6.0 software. Furthermore, the key genes were identified with molecular complex detection (MCODE) in Cytoscape for finding modules in PPI network.

#### 2.8.3 Validation of hub genes

Oncomine database (http://www.oncomine.org) was employed to detect the expression levels of RAD51AP1 in normal group and OC group. Kaplan‐Meier plotter (http://www.kmplot.com) was used to estimate the prognosis of 1435 OC patients. The OC patients were divided into two groups according to the median expression levels of RAD51AP1.

#### 2.8.4 Implementation of single‐sample gene set enrichment analysis (ssGSEA)

Marker genes set for immune cell types were extracted from Charoentong et al’s research.^13^ The infiltration levels of immune cell types were quantified by ssGSEA via ‘gsva’ package in R 3.5.0 software. The 28 immune cells included 12 anti‐tumour cells: activated CD4 T cell, activated CD8 T cell, central memory CD4 T cell, central memory CD8 T cell, effector memory CD4 T cell, effector memory CD8 T, type 1 T helper (Th1) cell, type 17 T helper (Th17) cell, activated dendritic cell, CD56 bright natural killer cell, natural killer cell, natural killer T cell; 8 pro‐tumour cells: type 2 T helper (Th2) cell, regulatory T cell, CD56^dim^ natural killer cell, immature dendritic cell, macrophage, neutrophil, plasmacytoid dendritic cell; and 8 other cells: active B cell, eosinophil, gamma delta T cell, immature B cell, mast cell, memory B cell, monocyte, T follicular helper cell. The correlation between risk score and ssGSEA score was calculated by Pearson correlation analysis.

#### 2.8.5 Gene Set Enrichment Analysis (GSEA) and Gene Set Variation Analysis (GSVA)

To investigate RAD51AP1‐mediated biological parameters in OC, GSEA was conducted by clusterProfiler package.^12^ We downloaded h.all.v6.2.symbols.gmt from the Molecular Signatures Database (http://www.broad.mit.edu/gsea/msigdb/). False‐discovery rate (FDR) < 0.05 and *P* < 0.05 were utilized as the enriched terms. In addition, 308 OC patients in the TCGA data set were divided into high‐expression group and low‐expression group according to the median value of RAD51AP1. GSVA conducted by ‘gsva’ package was applied to further validate different biological procedures between the two groups.

#### 2.8.6 Construction of a prognostic nomogram

It should be noted that OC patients have typically a poor prognosis; thus, we established a nomogram for evaluating the prognostic risk of each patient. TCGA data set was used to construct a nomogram using Cox proportional hazards model. The discrimination of the model was assessed by using the Harrell's concordance index (C‐index). Calibration curves were utilized to validate the accuracy of prediction. Diagnostic prediction models were analysed by receiver operating characteristic (ROC) curve and evaluated by the area under ROC curve (AUROC). The nomogram function was used in ‘rms’ package.

All the statistical analyses were performed using R 3.5.0 and GraphPad Prism 7.0 software. In at least three independent experiments, the data were presented as mean ± standard deviation (SD). Two‐tailed Student's t test was used to assess the differences between the two groups. *P* < 0.05 was considered statistically significant. All the data sets used in the present research are summarized in Table S2.

## 3 RESULTS

### 3.1 Identification and enrichment analysis of the DEGs

**FIGURE 1:**
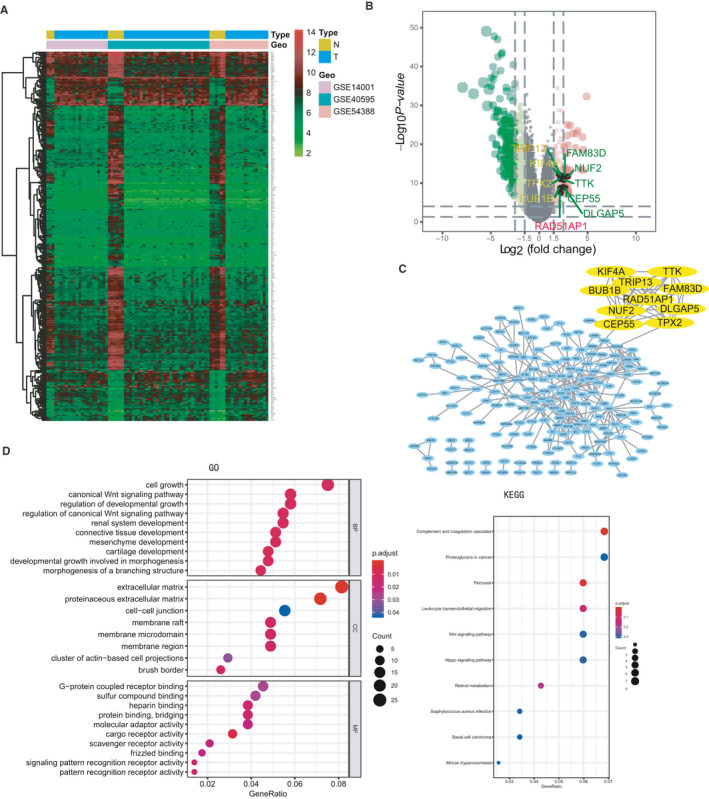
DEGs identified in GSE14001, GSE40595 and GSE54388. A, Heatmap of the 347 DEGs according to the selection criteria: adjusted *P* < 0.05 and |log FC|> 2. B, Volcano plot of DEGs between ovarian cancer and normal ovarian tissues. Dots with red colours represent up‐regulated DEGs, and green colours show down‐regulated DEGs. 10 hub genes were marked. C, PPI network of DEGs according to the inclusion criteria: combined score >0.4. 10 candidate hub genes were identified using the MCODE in Cytoscape: RAD51AP1, FAM83D, KIF4A, TTK, BUB1B, TRIP13, NUF2, CEP55, TPX2 and DLGAP5. D, Visualization of GO and KEGG analyses of the DEGs. Note: N: normal, T: tumour

A total of 347 DEGs were identified, including 75 up‐regulated genes and 272 down‐regulated genes in OC (Figure 1A and B). Subsequently, we constructed PPI network of DEGs, and one module was obtained using the MCODE in Cytoscape. Finally, 10 candidate hub genes were identified as follows (Figure 1C): RAD51AP1, FAM83D, KIF4A, TTK, BUB1B, TRIP13, NUF2, CEP55, TPX2 and DLGAP5. Their expression levels were all higher in OC group than those in control group in 3 GEO data sets (Figure S1).

To further analyse the biological functions and signalling pathways of the 347 DEGs, GO and KEGG enrichment analyses were undertaken. GO analysis mainly concentrated on cell growth, mesenchyme development, extracellular matrix, cell‐cell junction, protein binding, etc KEGG pathway indicated proteoglycans in OC, basal cell carcinoma, etc (Figure 1D).

### 3.2 Up‐regulation of RAD51AP1 was associated with poor survival in OC

**FIGURE 2:**
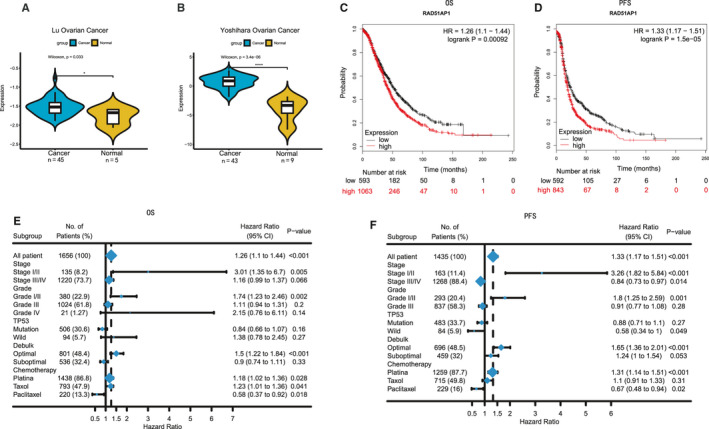
A and B, The expression level of RAD51AP1 was higher in OC patients based on Oncomine database. C and D, Patients with higher RAD51AP1 had poor overall survival (OS) and progression‐free survival (PFS) in Kaplan‐Meier plotter. E and F, Survival associated with RAD51AP1 in different subgroups (grade, stage, etc) in OC patients

RAD51AP1 is a critical protein for DNA repair by homologous recombination (HR). We analysed the expression level of RAD51AP1 in OC group and control group in Oncomine database, which revealed that RAD51AP1 was higher in OC (Figure 2A and B). Other types of cancer also showed similar results, in which the level of RAD51AP1 was higher in cancers, such as gastric cancer, cervix cancer, colorectal cancer and breast cancer (Figure S2A‐H).

We extracted the follow‐up data of RAD51AP1 in 1435 OC patients using Kaplan‐Meier plotter, in which up‐regulation of RAD51AP1 was found to be associated with poor OS and PFS (Figure 2C and D). Subgroup analyses revealed that at the early stage or grade, patients with higher RAD51AP1 presented as poor survival (Figure 2E and F).

### 3.3 High level of RAD51AP1 was associated with more mutational event in OC patients

**FIGURE 3:**
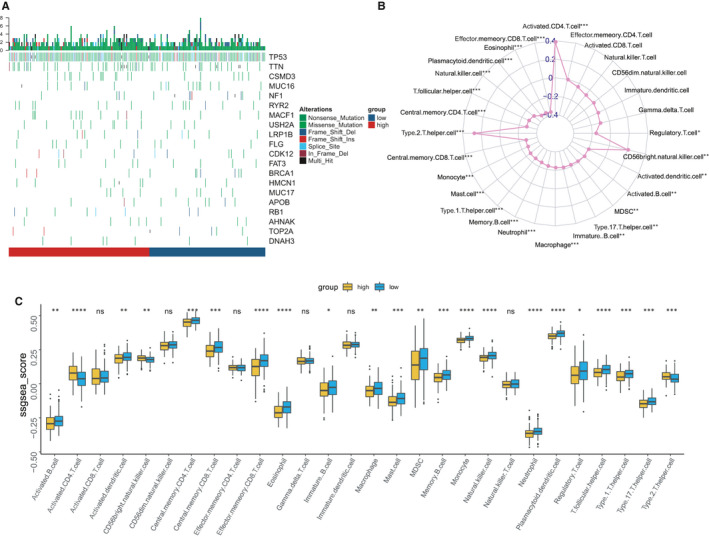
A, Gene mutations in OC subsets stratified by the expression level of RAD51AP1. More mutation burdens could be found in high‐RAD51AP1 group. B, Interaction of the TME immune cell types and RAD51AP1 based on TCGA data set in the radar chart. C, Tumour‐infiltrating cell level in high‐level RAD51AP1 group vs. low‐level RAD51AP1 group was estimated using the ssGSEA algorithm in TCGA

Previous genetic profiling studies have provided an accurate landscape of the driver gene mutations in OC.^14^ Based on these facts, we attempted to assess the correlation of RAD51AP1 expression with distinct mutational profiles of OC. The status of somatic mutation for OC was analysed by using ‘maftools' package. The top 20 mutated genes were TP53, TTN, CSMD3, MUC16, NF1, RYR2, MACF1, USH2A, LRP1B, FLG, CDK12, FAT3, BRCA1, HMCN1, MUC17, APOB, RB1, TOP2A, DNAH3 and COL6A3 in OC. In the TCGA OC RNA‐seq data set, 308 patients were initially divided into 2 groups based on the median value of RAD51AP1. Each group had 154 patients. However, only the patients who have the somatic mutation status data could be further analysed. Actually, there are 115 samples (74.7%) in high‐RAD51AP1 group, and 95 samples (61.7%) in low‐RAD51AP1 group with the somatic mutation status. TP53 was more mutated in both high‐RAD51AP1 group and low‐RAD51AP1 group. Other mutations of the 20 mutated genes emerged in high‐RAD51AP1 group, with a limited number of mutations in low‐RAD51AP1 group (Figure 3A). We suspected that mutations could accelerate progression of OC accompanied by increasing RAD51AP1 level.

### 3.4 Immune phenotype landscape of the tumour cells in the TME of OC samples

We evaluated the abundance of 28 tumour‐infiltrating immune cells in OC using TCGA data sets. In the radar chart, RAD51AP1 was statistically significant correlation with TME cells (Figure 3B). Then, the OC were divided into 2 clusters based on the median expression of RAD51AP1. The anti‐tumour cells including central memory CD4 T cell, central memory CD8 T cell, Th1 cell, Th17 cell, etc were higher in low‐level RAD51AP1 group; however, Th2 cell and the pro‐tumour cell were enriched in high‐level RAD51AP1 group (Figure 3C).

### 3.5 RAD51AP1‐related prognostic nomogram

**FIGURE 4:**
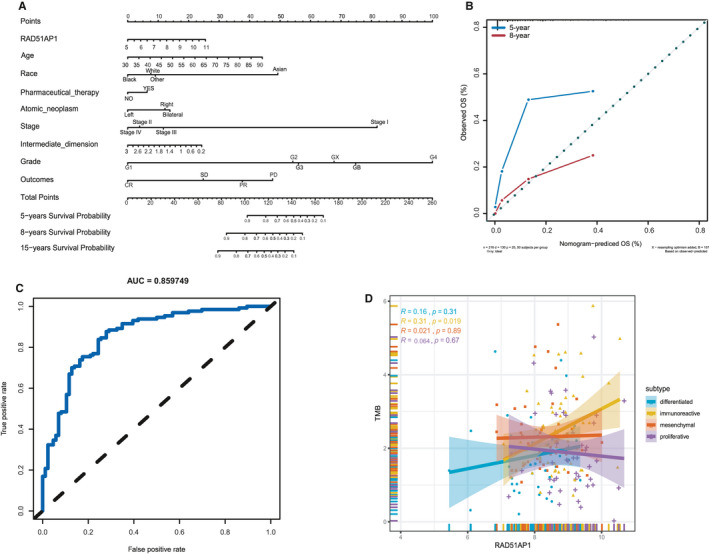
Establishment of the prognostic nomogram and investigation of relationship between RAD51AP1 and TMB. A, Nomogram for predicting OS based on clinical characteristic and RAD51AP1 in OC patients. The C‐index of the nomogram was 0.76. B, Calibration plot showed that predictive capability of 8‐year nomogram was notably superior than 5‐year one; C, ROC curve of RAD51AP1 in OC group (AUROC = 0.859749) for predicting OS. D, RAD51AP1 was positive with TMB

A prognostic nomogram that integrated variables of TCGA data set was constructed to predict the OS of OC patients (Figure 4A), including RAD51AP1 and several significant clinical factors, such as age, pharmaceutical therapy, atomic neoplasm, stage, grade, intermediate dimension and outcome. Calibration curves depicted that the predictive capability of 8‐year nomogram was remarkably superior than 5‐year one (Figure 4B). In our nomogram, RAD51AP1 was used as a new marker to increase the accuracy of model. The C‐index of the model was 0.76. ROC curve analysis of RAD51AP1 and clinical factors further demonstrated their accuracy in predicting OS of OC patients (AUROC = 0.859749, Figure 4C).

### 3.6 RAD51AP1 was positively correlated with tumour mutational burden (TMB)

It is noteworthy that TMB was recently deemed as an important marker to assess the reaction of patients to the target medicine (eg PD‐1/PD‐L1). It has been recommended as a useful marker for non‐small cell lung cancer (NSCLC) patients who underwent immunotherapy as described previously.^15^ The somatic mutation data of OC patients were processed to acquire 436 TMB data sets of OC patients. Besides, TMB and RAD51AP1 expression were simultaneously existed in 210 OC patients. Spearman's rank‐order correlation was used to explore the correlation of TMB with RAD51AP1, in which the scatter plots showed that TMB was correlated with increased RAD51AP1 (*P* < 0.05, Figure S3). Furthermore, in different four subgroups, TMB was significantly positively correlated with RAD51AP1 in immunoreactive group (Figure 4D). This might be a new outcome related to RAD51AP1 that the deserving gene assist scholars and clinicians to assess the efficacy of immunotherapy on OC patients.

### 3.7 RAD51AP1 accelerated proliferation of OC cells

**FIGURE 5:**
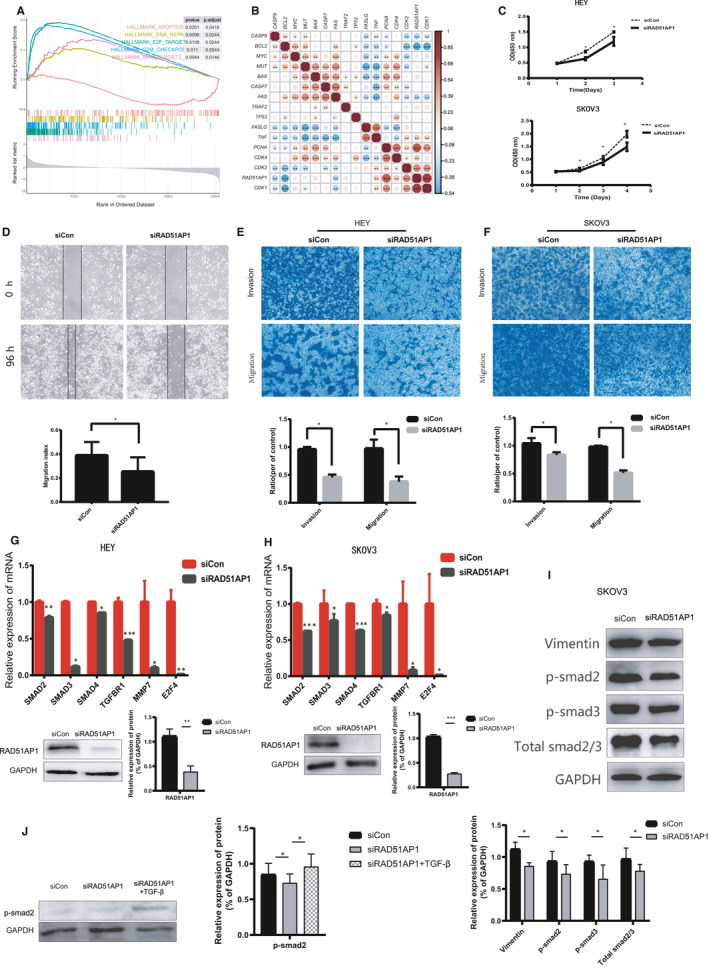
RAD51AP1 might accelerate proliferation, migration and invasion of OC cells by TGF‐β/Smad pathway. A, Enriched pathways found by Gene Set Enrichment Analysis (GSEA) using MsigDB: G2M Checkpoint, E2F Targets, MYC Targets and DNA Repair were enriched in OC samples with high expression level of RAD51AP1; apoptosis was enriched in OC samples with low‐expression level of RAD51AP1. B, Correlated heatmap illustrated that RAD51AP1 was positive associated with genes related to cell cycle, but negative associated with genes related to apoptosis. C, Down‐regulation of RAD51AP1 inhibited SKOV3 and HEY proliferation. D, Migratory distances of the siRAD51AP1 group were significantly wider. E and F, Migratory and invasive cells were dramatically reduced in SKOV3 and HEY transfected with siRAD51AP1. Compared to control cells, expression of SMAD2, SMAD3, SMAD4, TGFBR1 were lower in siRAD51AP1 cells in mRNA level (G and H) and protein level (I). J, Compared to control cells, expression of p‐smad2 in siCon group, siRAD51AP1 group and siRAD51AP1 + TGFβ group

GSEA shown that high‐RAD51AP1 group were mainly associated with G2M Checkpoint, E2F Targets and DNA Repair (Figure 5A), which were validated by GSVA (Figure S4). Additionally, in the correlation heatmap, cell cycle‐related genes were clustered and their expressions were positively correlated with RAD51AP1; besides, apoptosis‐associated genes were agminated and negatively correlated with RAD51AP1 (Figure 5B). These results demonstrated that RAD51AP1 was associated with cell cycle progression and accelerated cell proliferation.

To detect the effects of RAD51AP1 knockdown on cell viability, three distinct RAD51AP1 target‐specific siRNAs were used to transfect SKOV3. After 24 hours, the knockdown efficiency was validated by RT‐qPCR (Figure S5). Additionally, it was disclosed that siRAD51AP‐2 and siRAD51AP‐3 were more effective; consequently, the cells transfected with siRNA‐2 and siRNA‐3 were used for subsequent assays.

We further investigated the effects of siRAD51AP1 on cell proliferation. We found that down‐regulation of RAD51AP1 notably decreased the proliferation of SKOV3 and HEY (Figure 5C).

### 3.8 RAD51AP1 promoted the invasion and migration of OC cells

As shown in Figure 5D‐F, the capabilities of cell invasive and migration were lower in siRAD51AP1 cells than those in the control cells (*P* < 0.05). The wound healing assay showed that migratory distances of the siRAD51AP1 group were significantly wider (Figure 5D), reflecting that siRAD51AP1 inhibited SKOV3 migration. Transwell migration and invasion assays also clarified that migratory and invasive cells were notably decreased in SKOV3 (Figure 5E) and HEY (Figure 5F) transfected with siRAD51AP1.

### 3.9 RAD51AP1 accelerated progression of OC by TGF‐β/Smad signalling pathway

Bioinformatics analysis and in vitro assays demonstrated that RAD51AP1 accelerated progression of OC. To explore the mechanism, we studied the relationship between RAD51AP1 and genes related to TGF‐β/Smad signalling pathway in GEPIA (http://gepia.cancer‐pku.cn). Scatter plots showed that SMAD2, SMAD3, SMAD4 and TGFBR1 were significantly positively correlated with RAD51AP1 (Figure S6). Moreover, the results of RT‐qPCR and Western blotting were consistent with the scatter plots. Furthermore, mRNA levels of SMAD2, SMAD3, SMAD4 and TGFBR1 were lower in silent RAD51AP1 group (Figure 5G and H); besides, the protein levels of p‐SMAD2, p‐SMAD3 and total‐SMAD2/3 were lower in silent RAD51AP1 group (Figure 5I). In addition, when the cells were transfected with siRNA then incubated with TGFβ, p‐smad2 was lower in siRAD51AP1 group, but higher in siRAD51AP1 + TGFβ group compared to siControl group, indicating that TGFβ reversed the effect of siRAD51AP1 inhibiting p‐smad2 (Figure 5J).

## 4 DISCUSSION

OC accompanies by an increased incidence and poor survival in women. However, the evolutionary mechanisms of OC have still remained largely unknown. In the present study, we utilized the popular microarray and bioinformatics technologies and identified RAD51AP1 as a pivotal gene in 3 GEO data sets of 15 normal and 68 ovarian cancer samples.

RAD51AP1 is a critical protein for DNA repair by homologous recombination (HR), acting as a late‐acting accessory factor of the RAD51 recombinase and knockdown of RAD51AP1 results in increase of sensitivity to DNA damaging agents and impaired HR.^11^ High expression of DNA repair genes have been reported to be associated with a metastatic cancer.^16^ Consistent with the role of RAD51AP1 in a metastatic cancer, elevated RAD51AP1 might down‐regulate microRNA hsa‐mir‐140‐3p in OC,^17^ leading to reduction of survival time of breast and OC patients,^18^ as well as promoting ICC development and progression.^19^ In the current research, we explored the OS and PFS of OC patients based on Kaplan‐Meier plotter, which revealed that higher RAD51AP1 associated with poorer survival of OC patients.

Previous studies reported that RAD51AP1 is dysregulated in various types of cancer.^18^, ^20^, ^21^ In the present study, we confirmed that there was an up‐regulation of RAD51AP1 in OC and other types of cancer in Oncomine database. Moreover, RAD51AP1 was higher in higher stages or/and grades in breast cancer.

To our knowledge, gene mutation plays substantial roles in development and progression of cancer cells. However, no study had investigated the relationship between RAD51AP1 and gene mutations. With analysis of the TCGA data set, we found that high‐RAD51AP1 group exhibited more mutations. TP53 was more mutated in both high‐level RAD51AP1 group and low‐level RAD51AP1 group. Other top 19 mutations mainly emerged in high‐level RAD51AP1 group, while few mutations appeared in low‐level RAD51AP1 group. This indicated the fact that increase of RAD51AP1 might accelerate progression of OC.

TME consisted of tumour cells and other surrounding non‐tumour cells, such as immune cells and fibroblasts,^22^ which played important roles on the progression of tumour cells.^23^, ^24^ TME could directly invade surrounding tissues or metastasize through blood and lymphatic vessels. Recent studies have revealed that tumour progression can result from imbalances between tumour progression and the host immune response.^25^ In present study, tumour‐infiltrating immune cells results manifested high‐RAD51AP1 samples were infiltrated in Th2 cells, which was considered as the pro‐tumour cell implicated in severe disease.^26^ Tumour infiltrating lymphocytes (TILs), including T cells, B cells and NK cells, that exhibited anti‐tumoural functions,^27^, ^28^ especially CD8 + and CD4 + T cells were infiltrated in low‐level RAD51AP1 group. A number of studies showed that memory T cells and TH1 cells were associated with prolonged survival, higher of which were accompanied with better outcome.^29^, ^30^, ^31^ Therefore, OC patients with high RAD51AP1 might possess a generally coordinated and immunosuppressive TME, possibly accelerating progression of OC.

To date, studies conducted on survival of OC patients have been based on Kaplan‐Meier estimator, with ignored of other factors. Therefore, we, in the present research, constructed a RAD51AP1‐related nomogram to investigate patients’ OS. In our model, RAD51AP1 served as an indicator to increase its precision and specificity. Therefore, our study provided further credible results that might assist scholars. However, the limitation of our model was that we did not examine serum RAD51AP1 levels in this cohort. Besides, it is highly advantageous to conduct a case‐control study to examine serum RAD51AP1 levels.

The PD‐1/PD‐L1 blockade has become a promising approach for treating multiple types of cancer. Numerous patients benefited from such treatments, while some patients experienced hyperprogression after immunotherapy.^32^ Recently, TMB has emerged as a helpful marker to assess the clinical outcome of immunotherapy in NSCLC patients.^15^ Based on the scatter plots, TMB was significantly positively correlated with RAD51AP1 in immunoreactive group. Thus, RAD51AP1 might assist clinicians to evaluate the efficacy of immunotherapy in OC patients. Combination of RAD51AP1 and TMB might raise the capability that TMB forecasted the efficacy of immunotherapy, thus the results would be more credible.

As above, up‐regulation of RAD51AP1 might contribute to the progression of OC. Furthermore, functional analysis was performed to explore the biological functions, which demonstrated that RAD51AP1 might involve in cell cycle and proliferation. In vitro assay disclosed that RAD51AP1 promoted cell proliferation. Chudasama et al’s results ^22^ were consistent with the findings of the present study. Moreover, capabilities of cell migration and invasion were also suppressed by siRAD51AP1 in OC. Wu et al reported that siRAD51AP1 suppressed cell metastasis in lung cancer.^21^ Chudasama et al expressed that RAD51AP1 was up‐regulated in lung cancer patients and associated with mTOR signalling pathway.^33^ Whether RAD51AP1 promoted progression of OC by another pathway has still remained elusive.

In the present research, we explored the relationship between RAD51AP1 and markers in TGF‐β/Smad signalling pathway. The transforming growth factor β (TGF‐β) could regulate a fascinating array of cellular processes, including cell proliferation, apoptosis, differentiation, migration, invasion and adhesion. A number of scholars demonstrated that different types of cancer progressed along with activating TGF‐β/Smad signalling pathway.^34^, ^35^ Scatter plots showed that RAD51AP1 was positively correlated with genes in TGF‐β/Smad signalling pathway. Our experiments uncovered that the levels of p‐SMAD2, p‐SMAD3 and total‐SMAD2/3 were lower in siRAD51AP1 group; when the cells were transfected with siRNA then incubated with TGFβ, p‐smad2 was lower in siRAD51AP1 group, but higher insiRAD51AP1 + TGFβ group compared to siControl group, indicating that TGFβ reversed the effect of siRAD51AP1 inhibiting p‐smad2 (Figure 5J). This suggested that RAD51AP1 might promote cell proliferation, invasion and migration by TGF‐β/Smad signalling pathway.

## 5 CONCLUSIONS

In summary, we performed a comprehensive analysis on RAD51AP1. We found that up‐regulation of RAD51AP1 was closely associated with poor outcome and more mutations in OC patients. RAD51AP1 might have effect on TME, thus expedited progression of OC. We presented a further accurate nomogram for predicting prognosis of OC. Additionally, we demonstrated a potential mechanism of RAD51AP1, involving in the carcinogenesis, proliferation and invasion of OC might via TGF‐β/Smad signalling pathway.

## CONFLICT OF INTEREST

The authors declare that they have no competing interests.

## AUTHORS’ CONTRIBUTION

Hongyu Zhao: Data curation (supporting); Formal analysis (supporting); Software (supporting); Writing‐original draft (supporting). Yan Gao: Formal analysis (equal); Investigation (lead). Qi Chen: Formal analysis (equal); Investigation (equal). Jie Li: Formal analysis (equal); Methodology (equal). Meng Ren: Investigation (equal); Methodology (equal). Xiaoting Zhao: Validation (equal); Visualization (equal). Wentao Yue: Funding acquisition (supporting); Validation (supporting); Visualization (supporting); Writing‐review & editing (lead). HYZ, YG and WTY contributed to the conception and design of the study. HYZ drafted the manuscript and WTY revised the manuscript. QC verified the numerical results by an independent implementation. JL helped with the statistical analysis. RZ, DZ, MR and XTZ prepared all the figures and tables. All authors read and approved the final manuscript.

## Supporting information

Supplementary Material

## Data Availability

All the data sets used in the present research are summarized in Table S2.
