# Supplementary material for: RAD51AP1 promotes progression of ovarian cancer via TGF‐β/Smad signalling pathway
Source: J Cell Mol Med. 2020 Dec 13;25(4):1927–38. doi: 10.1111/jcmm.15877 (PMC7882964; doi:10.1111/jcmm.15877)
Supplement: Supplementary file 1 — Supplementary Material [file JCMM-25-1927-s001.docx]

**RAD51AP1 promotes progression of ovarian cancer via TGF-β/Smad signaling pathway**

Hongyu Zhao^1^, Yan Gao^1^, Qi Chen^1^, Ji Li^1^, Meng Ren^1^, Xiaoting Zhao^1#^, Wentao Yue^1#^

1 Central Laboratory, Beijing Obstetrics and Gynecology Hospital Capital Medical University, Capital Medical University, Beijing 100026, China

^#^ Corresponding authors.

E-mail address: yuewt@ccmu.edu.cn (WTY), zhao_xiaoting@126.com (XTZ)

Hongyu Zhao and Yan Gao contribute equally to the manuscript.

**Figure S1.** Heatmap of candidate 10 genes between ovarian cancer subjects and normal groups in three datasets.


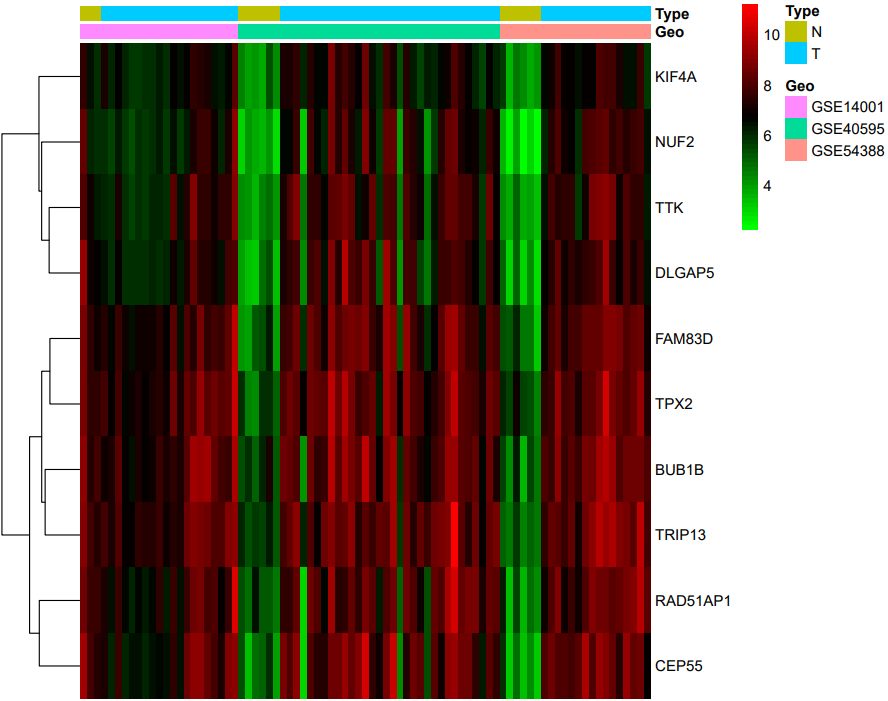
Note: N: normal, T: tumor.

**Figure S2.** Expression of RAD51AP1 in different cancers (A-G) and different grade (H) or stage (I) in breast cancer.


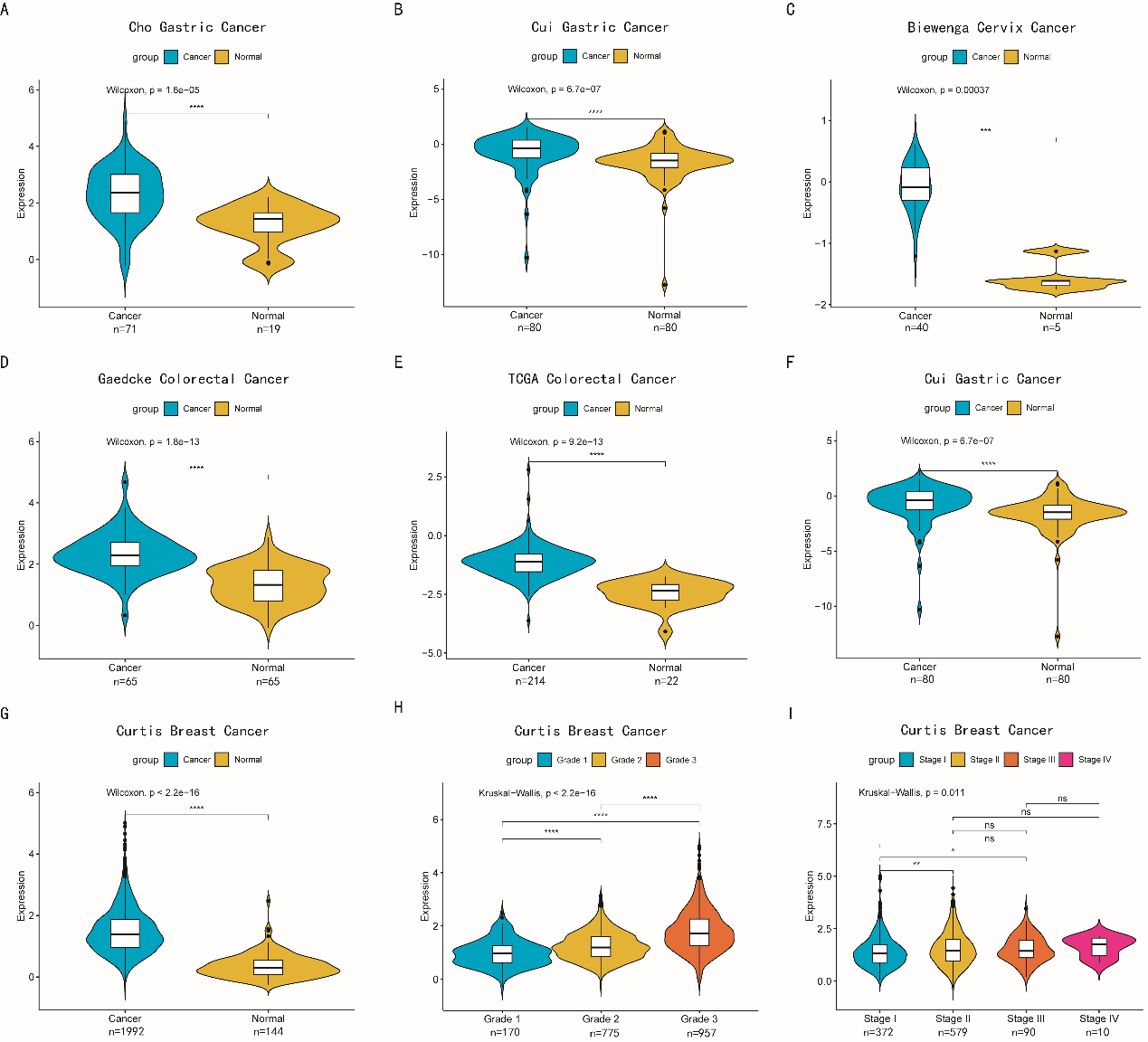
**Figure S3.**Scatter plot of TMB and RAD51AP1


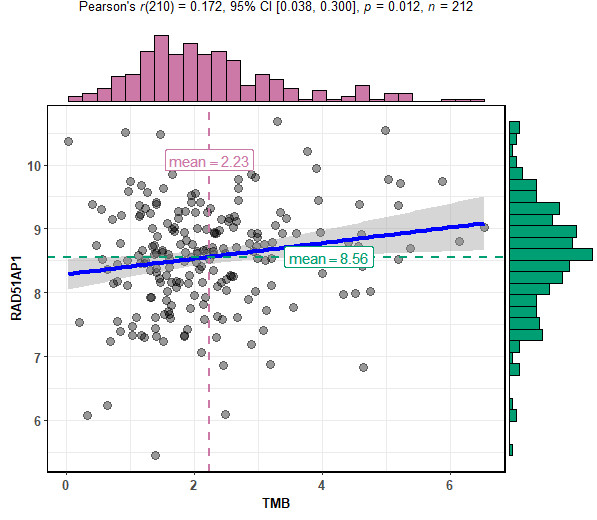


**Figure S4.** Enriched pathways found by Gene Set Variation Analysis (GSVA).


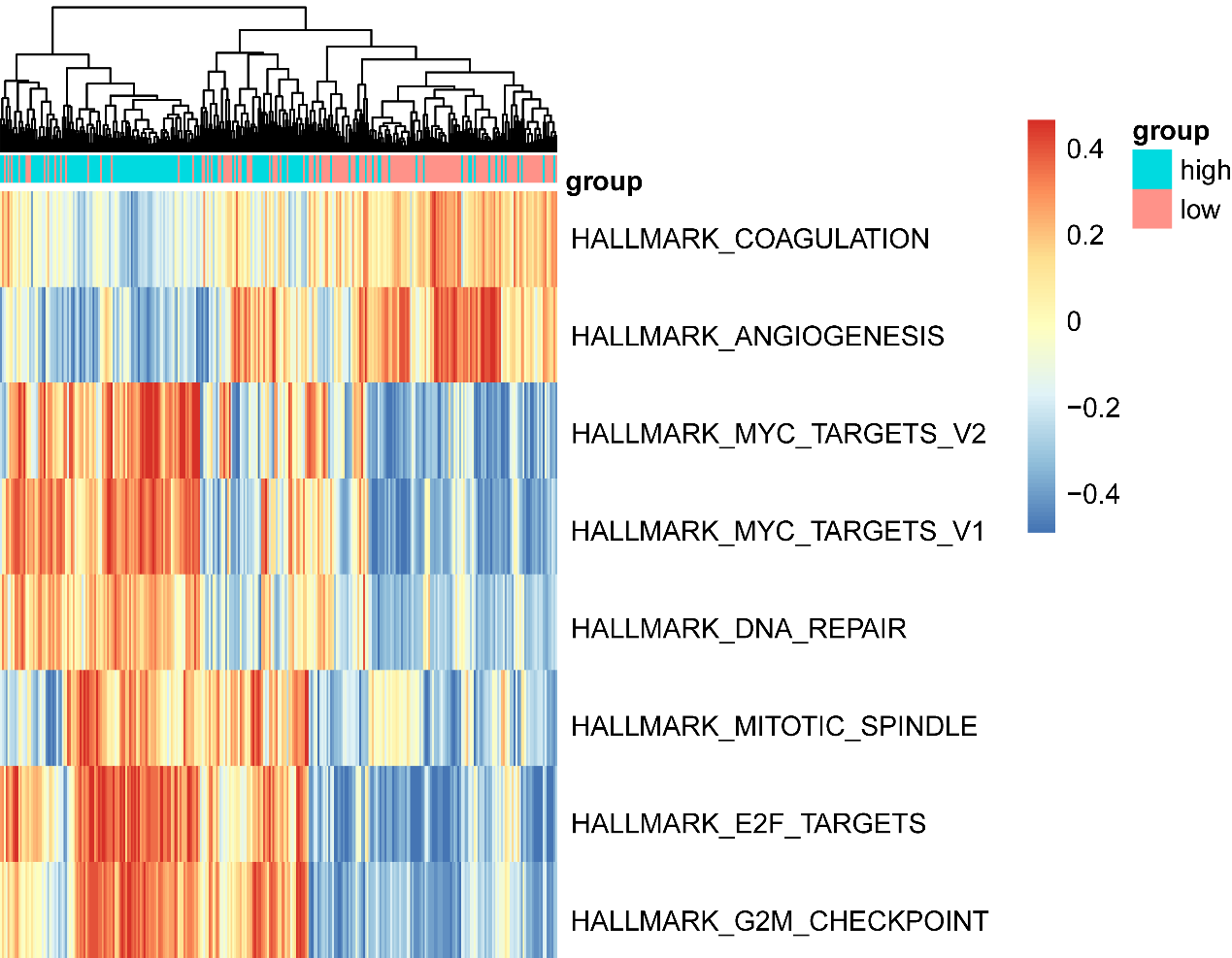


**Figure S5.** QRT-PCR analysis of siRAD51AP1 and siCon in SKOV3. siRAD51AP1 1 to 3 were all lower than siCon in SKOV3; siRAD51AP1 2 and 3 were lower compared to siRAD51AP1 1.

**Figure S6.** Scatter plots of RAD51AP1 and genes in TGF-β/Smad pathway.


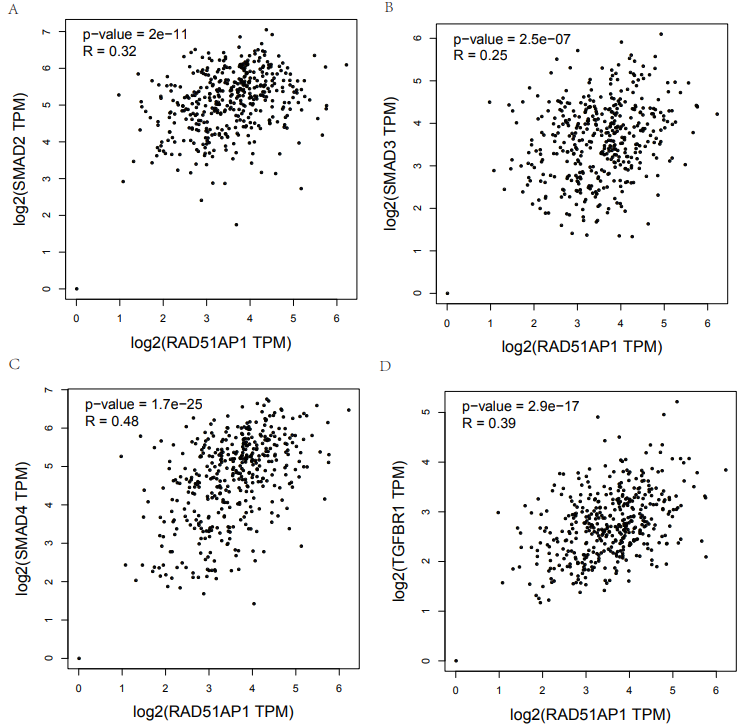


Table S1. Gene primer sequence

| Gene symbol | Primer sequence (5'--3') |
| --- | --- |
| RAD51AP1-F | ATGACAAGCTCTACCAGAGAGAC |
| RAD51AP1-R | CACATTAGTGGTGACTGTTGGAA |
| SMAD2-F | CGTCCATCTTGCCATTCACG |
| SMAD2-R | CTCAAGCTCATCTAATCGTCCTG |
| SMAD3-F | TGGACGCAGGTTCTCCAAAC |
| SMAD3-R | CCGGCTCGCAGTAGGTAAC |
| SMAD4-F | CTCATGTGATCTATGCCCGTC |
| SMAD4-R | AGGTGATACAACTCGTTCGTAGT |
| TGFBR1-F | ACGGCGTTACAGTGTTTCTG |
| TGFBR1-R | GCACATACAAACGGCCTATCTC |
| E2F4-F | CACCACCAAGTTCGTGTCCC |
| E2F4-R | GCGTACAGCTAGGGTGTCA |
| MMP7-F | GAGTGAGCTACAGTGGGAACA |
| MMP7-R | CTATGACGCGGGAGTTTAACAT |

Table S2. All the datasets used in the manuscript.

1. The Gene Expression Omnibus (GEO) OC datasets

| Platform | GSE ID | Samples | Download |
| --- | --- | --- | --- |
| Affymetrix HG-U133 Plus 2.0 (GPL570) | GSE14001 | 3 normal samples and 20 OC samples | GEO database (https://www.ncbi.nlm.nih.gov/) |
| Affymetrix HG-U133 Plus 2.0 (GPL570) | GSE40595 | 6 normal samples and 32 OC samples | GEO database (https://www.ncbi.nlm.nih.gov/) |
| Affymetrix HG-U133 Plus 2.0 (GPL570) | GSE54388 | 6 normal samples and 16 OC samples | GEO database (https://www.ncbi.nlm.nih.gov/) |

1. The Cancer Genome Atlas (TCGA) OC datasets

| Data | Samples | Download |
| --- | --- | --- |
| RNA-seq | 308 | UCSC Xena (https://xenabrowser.net/datapages/) |
| Somatic mutation status | 436 | GDC (https://portal.gdc.cancer.gov/repository) |

1. Kaplan-Meier plotter OC datasets

| Samples | Survival | Download |
| --- | --- | --- |
| 1435 | PFS | http://www.kmplot.com |
| 1656 | OS | http://www.kmplot.com |

1. GEPIA OC datasets

| Samples | Download |
| --- | --- |
| 426 | http://gepia.cancer-pku.cn |

1. Oncomine datasets

| Data | Samples | Download |
| --- | --- | --- |
| Lu Ovarian cancer | 5 normal samples and 45 tumor samples | http://www.oncomine.org |
| Yoshihara Ovarian cancer | 9 normal samples and 43 tumor samples | http://www.oncomine.org |
| Cho Gastric cancer | 19 normal samples and 71 tumor samples | http://www.oncomine.org |
| Cui Gastric cancer | 80 normal samples and 80 tumor samples | http://www.oncomine.org |
| Biewenga Cervix cancer | 5 normal samples and 40 tumor samples | http://www.oncomine.org |
| Gaedcke Colorectal cancer | 65 normal samples and 65 tumor samples | http://www.oncomine.org |
| TCGA Colorectal cancer | 22 normal samples and 214 tumor samples | http://www.oncomine.org |
| Curtis Breast cancer | 144 normal samples and 1992 tumor samples | http://www.oncomine.org |
| Curtis Breast cancer | 372 stage I samples, 579 stage II samples, 90 stage III samples, 10 stage IV samples | http://www.oncomine.org |
| Curtis Breast cancer | 170 grade I samples, 775 grade II samples, 957 grade III samples | http://www.oncomine.org |
